# EQ-5D in skin conditions: an assessment of validity and responsiveness

**DOI:** 10.1007/s10198-014-0638-9

**Published:** 2014-10-31

**Authors:** Yaling Yang, John Brazier, Louise Longworth

**Affiliations:** 1 Nuffield Department of Primary Care Health Sciences, University of Oxford, New Radcliffe House, Walton Street, Oxford, OX2 6NW UK; 2 Health Economics and Decision Science, School of Health and Related Research, University of Sheffield, Sheffield, UK; 3 Health Economics Research Group, Brunel University, Middlesex, UK

**Keywords:** EQ-5D, Validity, Responsiveness, Skin conditions, I120

## Abstract

**Aims and objectives:**

This systematic literature review aims to assess the reliability, validity and responsiveness of three widely used generic preference-based measures of health-related quality of life (HRQL), i.e., EQ-5D, Health Utility Index 3 (HUI3) and SF-6D in patients with skin conditions.

**Methods:**

A systematic search was conducted to identify studies reporting health state utility values obtained using EQ-5D, SF-6D, or HUI3 alongside other HRQL measures or clinical indices for patients with skin conditions. Data on test-retest analysis for reliability, known group differences or correlation and regression analyses for validity, and change over time or responsiveness indices analysis were extracted and reviewed.

**Results:**

A total of 16 papers reporting EQ-5D utilities in people with skin conditions were included in the final review. No papers for SF-6D and HUI3 were found. Evidence of reliability was not found for any of these measures. The majority of studies included in the review (12 out of 16) examined patients with plaque psoriasis or psoriatic arthritis and the remaining four studies examined patients with either acne, hidradenitis suppurativa, hand eczema, or venous leg ulcers. The findings were generally positive in terms of performance of EQ-5D. Six studies showed that EQ-5D was able to reflect differences between severity groups and only one reported differences that were not statistically significant. Four studies found that EQ-5D detected differences between patients and the general population, and differences were statistically different for three of them. Further, moderate-to-strong correlation coefficients were found between EQ-5D and other skin-specific HRQL measures in four studies. Eight studies showed that EQ-5D was able to detect change in HRQL appropriately over time and the changes were statistically significant in seven studies.

**Conclusions:**

Overall, the validity and responsiveness of the EQ-5D was found to be good in people with skin diseases, especially plaque psoriasis or psoriatic arthritis. No evidence on SF-6D and HUI3 was available to enable any judgments to be made on their performance.

## Introduction

In the UK and elsewhere, a common practice in economic evaluation of health technologies is to use cost-utility analysis, where results are presented as an incremental cost per quality adjusted life year (QALY) gained [1, 2]. The QALY method provides a way of measuring the benefits of health care interventions by combining both improvements in health-related quality of life (HRQL) and extension of life years into a single index. The QALY is estimated by weighting survival with a value reflecting the HRQL experienced during the time period, where the HRQL value is estimated to reflect the ‘utility’ of the health state.

Health state utility values are commonly estimated using one of the generic preference-based measures (GPBMs) of HRQL. Examples of the most commonly used GPBMs include the EQ-5D [3, 4], SF-6D [5] and the Health Utilities Index (HUI) [6]. GPBMs typically use a multi-dimensional, multi-level descriptive system of health combined with a utility value set that can be applied to each unique health state described by the system. The health state utility values associated with GPBMs are usually obtained from general population-based valuation studies using techniques such as time trade-off or standard gamble. These values are on a scale where a weight of 0 corresponds to a health state ‘dead’ (as well as, potentially, to health states considered as bad as being dead) and a weight of 1 corresponds to full health, which meets the requirement for QALY calculation. The combination of the generic descriptive system and value sets of GPBMs enables users to reflect the value people place on different health states make comparisons of health outcomes across different conditions [7].

The descriptive systems of the commonly used GPBMs differ in terms of their dimensions. EQ-5D has five dimensions of health including mobility, self-care, usual activities, pain/discomfort, and depression/anxiety. The original version of EQ-5D has three levels of severity in each dimension; and a version with five levels of severity has recently been developed [8]. The three-level version describes 243 health states. We refer to the three level EQ-5D though out this paper. Derived from the SF-36 and SF-12 health questionnaires, the SF-6D has six dimensions of health including physical functioning, role limitation, social functioning, bodily pain, mental health, and vitality, and each dimension has four to six severity levels. The health state of any patient who completes the SF-36 or the SF-12 can be classified according to the SF-6D system. The health classification system of SF-6D describes a total of 18,000 health states. The HUI3 has eight dimensions of health including vision, hearing, speech, ambulation, dexterity, emotion, cognition, and pain, and each dimension has five or six severity levels. The health classification system of HUI3 describes almost a million unique health states. The HUI3 can be seen as a ‘within-the-skin’ measure of health because it contains sensory dimensions such as vision, speech and hearing, and concerns health or health problems, whereas the EQ-5D and SF-6D focus more on how health impacts on functioning in life although both SF-6D and EQ-5D also have symptom-related dimensions (e.g., pain and discomfort).

Apart from the different descriptive systems, the sample populations, valuation, and extrapolation techniques used to arrive at the value sets of the measures also differ. Several value sets are available for the EQ-5D to reflect the different values of different countries such as UK, France, Germany, Netherlands, Denmark, Spain, Japan, and USA. The UK value set of EQ-5D has been the most widely used and it was obtained from valuations provided by 3,395 members of the general population using the time trade-off valuation method. The UK value set of SF-6D was obtained from valuations provided by 611 members of the general population using the standard gamble valuation method and similar values sets have been obtained in Japan, Hong Kong, Portugal, and Brazil. Similarly, tariffs of values for each health state of HUI3 is available estimated from Canadian and UK samples. The original Canadian value set was obtained from valuations provided by 504 members of the general population using the visual analogue scale (VAS) and standard gamble (SG) valuation methods.

Given the different descriptive systems and valuation methods, there has been evidence showing that health state utility values obtained from the three GPBMs can be different [9–11]. GPBMs, especially EQ-5D, have attracted criticism for perceived failure to capture important aspects of health and insensitivity to change in specific health conditions [12–16]. There might be specific circumstances in which the EQ-5D or other GPBMs are not appropriate to use. Therefore, it is important to assess the performance of EQ-5D and other GPBMs for a wide range of conditions and/or treatments. This type of research can provide evidence on whether these measures are appropriate for those specific conditions and aid the judgment of whether or when alternative measures should be considered. The examination of the validity and responsiveness of GPBMs is fraught with conceptual and empirical problems due to the lack of a gold standard measure. However, conventional psychometric tests of construct validity and responsiveness can inform judgments about the appropriateness of measures of health in a comprehensive and transparent way [17].

A review of the evidence on the psychometric performance of GPBMs in skin disorders has not been previously undertaken. On the other hand, skin disorders like psoriasis and atopic eczema have a profound influence on patients’ lives. The painful or itching symptoms of skin conditions may affect patients’ social lives, their daily work, and their personal relationships [18, 19]. The aim of this study was to systematically review the published literature to assess the reliability, validity, and responsiveness of three key generic measures of health-related quality of life (EQ-5D, HUI3, and SF-6D) in people with skin disorders.

## Methods

### Search strategy and data identification

We conducted a systematic search of published papers reporting EQ-5D, HUI3, and SF-6D in patients with skin diseases using a search strategy developed following consultation with experts in information resources, clinicians, and health economists. The search strategy focused on keywords, including ‘skin impairment/disorder/disease’, ‘euroqol/EQ-5D’, ‘hui3’, and ‘sf6d’, all with alternative spellings. Specific terms of skin diseases were obtained from ICD-10; examples, included ‘impetigo’ ‘furunculosis’, and ‘cutaneous abscess’. The search strategy used for MEDLINE is presented in the "Appendix".

We searched the following electronic databases: BIOSIS (1969–2010); CINAHL (1982–2010); Cochrane Library comprising the Cochrane Database of Systematic Reviews (CDSR), Cochrane Central Register of Controlled Trials (CENTRAL), Cochrane Methodology Register, NHS Economic Evaluations Database (NHS EED) (1991–2010); EMBASE (1980–2010); MEDLINE (in process and non-indexed–2010); PsychNFO (1806–2010); and Web of Science (1900–2010). We also conducted a search of the EuroQol Group database for possible relevant studies for EQ-5D [20]. Similar searches were not conducted for HUI3 and SF-6D as comparable databases are not available.

We used the following inclusion criteria to identify relevant papers, where:

1. the study population had any skin diseases; and
2. the study reported at least one of the three GPBMs (EQ-5D, SF-6D, or HUI3); and
3. the study reported another measure of quality of life (generic or condition-specific), a measure of clinical severity, or direct valuation of health.

This implies that papers are excluded if:

1. the study only reported EQ- Visual Analogue Scale (VAS) scores; or
2. the study only used vignettes or own health state valuations, not one of the three generic measures; or
3. the study did not report another measure of quality of life (generic or condition-specific) or a measure of clinical severity, or direct valuation of health, alongside the three measures of interests; lastly papers were excluded where they were written in languages other than English.

### Data extraction

We extracted data from the included studies using a form developed in Microsoft Excel, which covered general characteristics of the study and participants, instruments used in the study, methods and relevant results provided in the included study for an assessment of reliability, construct validity, and responsiveness. Studies did not have to be specifically designed to assess reliability, responsiveness or validity provided sufficient data were presented to allow us to make an assessment. For example, studies were included if they reported results of analyses of change over time using the GPBM and a comparison measure (to indicate a change had occurred) or if they reported analyses of the GPBM according to subgroups defined by a comparison measure of health (known group validity). Our analyses are based on data provided in the included papers and we did not carry out these analyses by ourselves. Data extraction was undertaken by one member of the research team and summarized using items presented in Table 1.

**Table 1:** Data extracted from included papers

General information of the study	Author name(s), publication year
	Country where study was conducted
	Type of skin diseases
	Disease/treatment stage
	Treatment (if any)
	Study design (e.g., randomized control trial, cross-sectional study, etc.)
Characteristics of participants	Number of participants
	Age (mean and range)
	Gender (percentage of males)
	Ethnicity
	Missing data (respondent drop-out/non-completion), including reasons for non-completion if given
Measures and valuations reported	General measures used
	Tariff or source of value sets
	Mean values reported (standard deviation range)
	Direct valuations used
	Condition-specific HRQL measures used
	Clinical measures used
	Qualitative questions asked
	Missing data (within measure completion)
Reliability	Methods used
	Results reported
Validity	Methods used (e.g., known group, correlation coefficients, regressions)
	Results reported
Responsiveness	Methods used (e.g., significance of change over time, effect size).
	Results reported

### Data analysis

#### Assessment of quality and relevance

The quality of a study was assessed by examining the risk of bias from the methods of patient recruitment, and noting any missing data reported either study drop-outs or incomplete questionnaires. The purpose of assessing study quality was not to exclude relevant studies, but to highlight any concerns about quality when findings were being interpreted. Most important was to assess the relevance of the study in terms of the patient population and evidence to judging the psychometric performance of the generic measures. Studies were not required to be specifically designed to assess validity, responsiveness or reliability provided that they reported data in a sufficient detail to allow an assessment of these.

#### Assessment of reliability

A measure can claim reliability if it reproduces stable results when measurements are repeated on an unchanged population. Reliability can be assessed by test-retesting and reporting the correlation or difference between estimates. Where GPBM values did not change over time and other measures of health demonstrated no change in health over the same period, the results were interpreted as evidence of the reliability of instruments.

#### Assessment of construct validity

Validity is defined as how well an instrument measures what it was intended to measure. Validity can be assessed by comparing an instrument to an established gold standard; however a gold standard does not exist in health utility measurement. Therefore it is necessary to assess the validity of GPBMs using measures having evidence of construct validity, which establishes if patterns in scores confirm constructs or hypotheses about expected patterns.

We assessed the construct validity of the GPBMs using the ‘known group’ method that compares (qualitatively or statistically using *t* test or ANOVA) the values obtained from the GPBMs between groups of patients who are expected to differ according to clinical severity or other measures of HRQL. Known groups can also be defined using a case–control analysis where comparison is between population of patients and the general public without the condition; or defined on the basis of other aspects such as age, gender, etc.

We also examined convergent validity, which is a type of construct validity. Convergent validity is defined as the extent to which one measure correlates with another measure of the same or similar concept. In this review, we examined the extent to which EQ-5D, SF-6D or the HUI3 correlate with other measures of HRQL or clinical severity. Correlation was defined as ‘low’ if the correlation coefficient was less than 0.3, ‘moderate’ if between 0.3 and 0.5, and ‘strong’ if greater than 0.5. Further, we interpreted regression estimates of the relationship between GPBMs and other measures as another indication of convergence focusing on whether measures were significant predictors of the others.

#### Assessment of responsiveness

Responsiveness assesses the ability of an instrument to measure a change in health over time. As with construct validity, there is no gold standard measure for change. We assessed the responsiveness of the GPBMs by comparing change in GPBM values over a period of time in which health status is expected to change (for example before and after an intervention) with the change demonstrated by another measure of health. We considered there to be strong evidence of responsiveness if the GPBM showed statistically significant change in health (e.g., *t* test), which was demonstrated by other measures or clinical indicators. Where there was the expected trend of change (e.g., improvement or decline) but the change was not statistically significant then this was interpreted as weak supportive evidence.

We also compared responsiveness indices (e.g., effect size or standard response mean) of health-related utility with those of other measures when they were reported. Effect size is the mean change score of a measure between two time points divided by the standard deviation of the score at baseline whereas standardized response mean is similarly the mean change score divided by the standard deviation of the change score [21].

## Results

### Search results

**Fig. 1:**
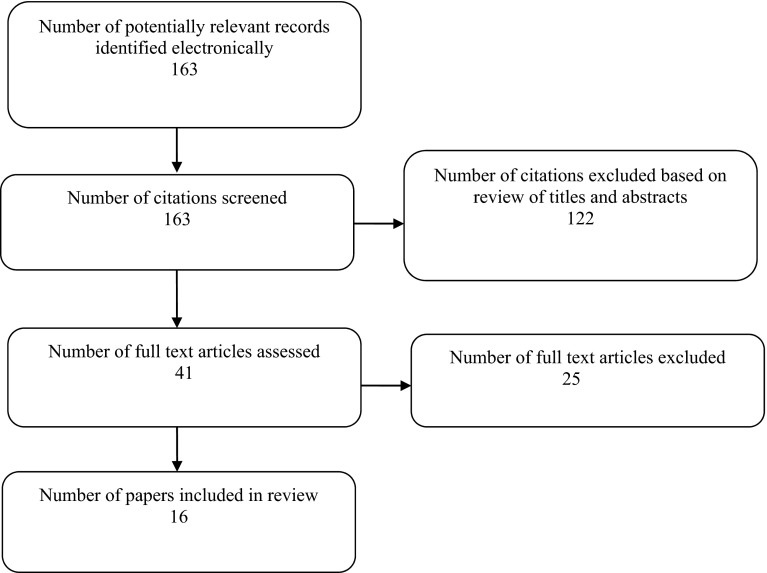
Flow diagram showing selection of studies for skin review

The bibliographic search identified a total of 161 records from the electronic databases and two additional records from the EuroQol Group website database. We excluded 122 records after reviewing titles and abstracts. Forty-one papers were reviewed in full, a further 25 papers were excluded and 16 papers were included in the final review (see Fig. 1).

### Quality assessment—skin conditions

The included studies reported three types of study designs. Eleven studies were RCTs (including one study only reported the baseline data), four studies were cross-sectional, and one was an uncontrolled before-and-after study. The majority of studies provided clear inclusion and exclusion criteria for recruitment of patients; however inclusion and exclusion criteria were not clear for two studies [22, 23]. Ten studies reported that between 70 and 97 % respondents completed the planned follow-up; data were not reported on completion for six papers. The completion rates of individual questionnaires (i.e., item response of a questionnaire with no missing data) were generally high (above 90 %). No study was excluded after the assessment of quality.

### Study design, patients’ characteristics, and measures used in studies

The main characteristics of the 16 papers included in this review are shown in Table 2. Studies were conducted in various European and American countries, with several multi-national studies. All but five studies recruited patients with psoriasis, the remaining studies recruited patients with acne, eczema, hidradenitis suppurativa, or venous leg ulcers. All studies included adult patients (mean age around 43 years). In these studies, male respondents accounted for 24–71 % of the samples. Sample size ranged from 32 to 27,994 but most studies had a sample size of between 100 and 200. EQ-5D utility values were reported in all but two studies and the mean values ranged from 0.5 to 0.82.

**Table 2:** Characteristics of studies and measures used

	Skin diseases	Study design	Treatment	Number of participants	Mean age (years)	Male (%)	EQ-5D mean values at baseline	Other measures used
Bansback et al. [24]	PsA	RCT	Methotrexate with and without ciclosporin A	72	47	44 (placebo) 29 (Ciclosporin)	0.52 (range, 0.48–1)	HAQ-DI
Brodszky et al. [30]	PsA	Cross-sectional	None	183	50	43	0.5 (SD 0.3)	PsAQoL, HAQ, PASI, DAS28, BASDAI, swollen joint count, tender joint count. EQ-VAS, patient pain VAS, patient global assessment VAS
Christophers et al. [25]	PP and PsA	Cross-sectional	N/R	1,660	46 (PP) 49 (PsA)	42 (psoriasis) 32 (PsA)	0.82 psoriasis 0.56 PsA	BSA, employment disadvantage questionnaires
Daudén et al. [26]	PP	RCT	Continuous vs. paused subcutaneously (SC) therapy	720	45 (continuous therapy); 45 (paused therapy)	38 (both therapy)	0.65 (continuous therapy); 0.66 (paused therapy)	DLQI, HADS, PSS, BSA, PGA, SF-36 vitality subscale, EQ-VAS
Van de Kerkhof [23]	PP	RCT	TCP (+vehicle once daily) TCP (twice daily), Calciogtriol (twice daily), Vehicle (twice daily)	828	49	36	N/R (EQ-5D responses reported)	PDI, EQ-VAS
Luger et al. [27]	PP	Randomized, open-label study	Continuous and paused etanercept therapy	720	46 (joint pain), 44 (no joint pain); 45.3 (nail psoriasis) 43.9 (no nail psoriasis)	31 (joint pain), 24 (no joint pain) 25 (nail psoriasis) 43 (no nail psoriasis)	0.59 (joint pain), 0.79 (no joint pain); 0.65 (nail psoriasis), 0.7 (no nail psoriasis)	DLQI, HADS, PASI, SGA, PGA, BSA, SF-36 vitality subscale, NAPSI, EQ-VAS
Reich et al. [28]	PP	RCT	Etanercept	142	45	58	0.66 (placebo) 0.70 (Etanercept)	DLQI, FACIT-F, PASI, BSA, EQ-VAS
Revicki et al. [32]	PP	RCT	Adalimumab, MTX, placebo	271	40 (placebo); 42 (MTX), 43 (adalimumab)	66 (placebo) 67 (MTX) 66 (Adalimumab)	0.7 (SD 0.2)	DLQI, PASI, VAS for pain VAS for psoriasis-related pruritus assessment
Shikiar et al. [31]	Psoriasis	RCT	Subcutaneously administered adalimumab vs. placebo	147	44	66	0.66	DLQI, PASI, PGA, EQ-VAS, SF-36
Shikiar et al. [33]	Psoriasis	RCT	Subcutaneously administered adalimumab vs. placebo	148	43 (placebo) 46 (Adalimumab)	65 (Placebo) 71 (Adalimumab)	0.67 (placebo); 0.69 (treatments)	DLQI, PASI, PGA, SF-36
Weiss et al. [29]	Psoriasis	RCT (baseline only)	N/R	35	49 (median)	60	0.72	PASI, SAPASI, SF-36, SWLS
Weiss et al. [34]	Psoriasis	RCT	Topical therapy vs. combination clobetasol solution	32	49 (median)	N/R	0.78	DLQI, PASI, SAPASI, BSA, EQ-VAS
Klassen et al. [22]	Acne	Before and after study	Isotretinoin or antibiotic, hormonal, physical and topical treatments	130	22	61	0.82 (SD 0.16)	DLQI, acne grade, EQ-VAS
Matusiak et al. [35]	Hidradenitis suppurativa	Cross-sectional	N/R	54	40	49	0.66	DLQI, BDI-SF, FACIT-F, QLES-Q, GQ, 6-item scale, Hurley’s classification, EQ-VAS
Moberg et al. [36]	Hand eczema	Cross-sectional	N/R	27,994	18–64	45	N/R (EQ-5D responses reported)	EQ-VAS
Walters et al. [37]	Venous leg ulcers	RCT	Compression bandaging in a community clinic setting vs. usual home-based care by district nursing services	233	75 (median)	34	0.57 (SD 0.18)	FAI, SF-MPQ Self-perceived transition question (item 2 of SF-36) with 3 scales: better, same and worse comparing to 3 months earlier, EQ-VAS, SF-36

*PsA* Psoriatic arthritis, *PP* Plaque psoriasis, *HAQ-DI* Health Assessment Questionnaire Disability Index, *PsAQoL* Psoriatic Arthritis Quality of Life scale, *HAQ* Health Assessment Questionnaire, *PASI* Psoriasis Area Severity Index, *DLQI* Dermatology Life Quality Index, *HADS* Hospital Anxiety and Depression Scale, *PSS* Patient Satisfaction survey, *NAPSI* Nail Psoriasis Severity Index, *BDI-SF* Beck Depression Inventory—short form, *FACIT-F* Functional Assessment of Chronic Illness Therapy—Fatigue scale, *Q-LES-Q* Quality of Life Enjoyment and Satisfaction Questionnaire—Short Form, *GQ* Global question index, *FAI* the Frenchay Activities Index, *SF-MPQ* the McGill Short Form Pain Questionnaire, *DAS28* the 28 joint Disease Activity Score, *BASDAI* the Bath Ankylosing Spondylitis Disease Activity Index, *BSA* Body Surface Area affected with psoriasis, *PGA* Physician Global Assessment of psoriasis, *SAPASI* PASI presented in non professional terminology, *SGA* Subject Global Assessment for joint pain, *PGA* Physician Global Assessment of Psoriasis, *SWLS* Satisfaction With Life Scale

The measures used in the 16 studies are summarized in the last column of Table 2. Of the three GPBMs of interest, only EQ-5D data were found and included in the review. No studies reported data from SF-6D and HUI3. The majority of studies used the UK tariff to obtain the EQ-5D utility values but for several studies it was not clear which tariff was used [23–25]. Fourteen studies reported VAS scores of patients’ own perceived health in addition to the EQ-5D index values. Various clinical indices were reported to indicate severity of skin problems, including Psoriasis Area Severity Index (PASI) in eight studies, Nail Psoriasis Severity Index (NAPSI) in one study, and acne grade in one study. Several generic measures [e.g., SF-36, Health Assessment Questionnaire Disability Index (HAQ-DI), Health Assessment Questionnaire (HAQ)], skin-specific HRQL measures [e.g., Dermatology Life Quality Index (DLQI)], or symptom specific HRQL measures [e.g., Hospital Anxiety and Depression Scale (HADS); Depression Inventory] were reported in these studies.

### Reliability

No study reported data on reliability of the three GPBMs.

### Construct validity and responsiveness

Thirteen studies among patients with skin problems provided sufficient evidence to allow assessment of known group validity and convergent validity of EQ-5D. Among them, nine studies included patients with psoriasis or psoriatic arthritis, one study each included patients with acne, hidradenitis suppurativa, hand eczema, and venous leg ulcers.

Eleven studies among people with skin problems provided evidence to allow assessment of responsiveness of EQ-5D. Among them, eight studies included patients with psoriasis or psoriatic arthritis, one study included patients with acne, and one study focused on venous leg ulcers. Ten studies examined changes of scores over time or after treatment, and two provided details of effect size or standard response mean estimation. One study checked the correlation between change scores of health measures with changes in clinical measures.

We summarize findings of construct validity and responsiveness of EQ-5D on various skin conditions below.

### Plaque psoriasis and psoriatic arthritis

#### Known group analysis

Seven studies allowed known group analysis for EQ-5D among people with psoriasis or psoriatic arthritis. Among them, three studies showed that EQ-5D was able to discriminate severity groups significantly. Christopher et al. [25] reported that EQ-5D values of people with psoriatic arthritis (PsA) were statistically lower than psoriatic arthritis (0.56 vs. 0.82, *p* < 0.001). Daudén [26] reported that EQ-5D values differed between the two treatment groups (*p* < 0.05) and this was confirmed by EQ-VAS and DLQI but not HADS-Depression and HADS-Anxiety subscale, or SF-36 vitality and the satisfaction survey. Another study conducted by Luger [27] indicated that EQ-5D was able to discriminate (*p* < 0.1) between patients with or without joint pain, and patients with or without nail psoriasis, which was consistent with a series of measures including EQ-VAS, PASI, DLQI, SF-36 vitality, and HADS.

Three case–control studies confirmed that EQ-5D can differentiate between people with psoriasis and the general population [23, 28, 29]. Another study by Brodszky et al. [30] found that the standardized mean difference between groups measured by EQ-5D were lower than that produced by the Psoriatic Arthritis Quality of Life Instrument (PsAQoL) and the HAQ. However, the groups were defined according to admission to hospital, receipt of a disable pension, use of devices or requiring help from others for everyday activities; whilst these may be suggestive of disease severity they are likely to be confounded by other factors, for example, disabled pension maybe indicative of age or better overall income than those who receive a different kind of pension.

#### Convergent validity

Four studies provided evidence of convergent validity for EQ-5D among patients with psoriasis and psoriatic arthritis. Three studies showed moderate or strong correlation between EQ-5D and other generic or skin-specific measures. Brodsky et al. [30] reported a strong correlation coefficient of over 0.5 between EQ-5D and HAQ, Psoriatic Arthritis Quality of Life scale (PsAQoL), the pain VAS, the patient global VAS and the Bath Ankylosing Spondylitis Disease Activity Index (BASDAI). Shikiar et al. [31] found that EQ-5D was moderately to strongly correlated with EQ-VAS, DLQI, PASI, Physician Global Assessment of psoriasis (PGA), and SF-36 domains. Similarly, Weiss et al. [29] demonstrated that EQ-5D was strongly correlated with Patient’s Satisfaction With Life Scale (SWLS) scores (correlation coefficients 0.46, *p* < 0.05) and eight domains of SF-36 (correlation coefficients ranged from 0.62 to 0.78, *p* < 0.001). Through a regression analysis, Bansback et al. [24] suggested that the HAQ disability index was a significant predictor of EQ-5D (coefficient −0.31, *p* < 0.05).

#### Responsiveness

All nine studies among patients with psoriasis or psoriatic arthritis confirmed that EQ-5D was responsive to change in health over time in this condition. Daudén et al. [26] reported that being consistent with the EQ-VAS, DLQI, HADS-Anxiety scale, and the SF-36 vitality dimension, EQ-5D values improved significantly (*p* < 0.05) and clinically meaningfully from baseline for both treatment groups. Luger et al. demonstrated that EQ-5D values improved significantly (change of 0.17, by 29 %) alongside EQ-VAS (change of 12.87, by 23 %), DLQI (change of 8.86, by 61 %), the SF-36 vitality dimension (change of 5.6, by 11 %), HADS-Depression (change of 1.9, by 29 %), HADS-Anxiety (change of 2.27, by 28 %) among patients with joint pain. However, for patients with nail psoriasis, EQ-5D did not detect a significant improvement, whereas a significant improvement was found by other measures [27]. Reich et al. [28] reported that at both follow-up time points, the group who received active treatment achieved significant improvement compared to placebo measured using EQ-5D, EQ-VAS, FACT-Fatigue, and DLQI (both total and domain scores). Similarly, Revicki et al. [32] reported that statistically significant improvement (*p* < 0.001) was detected for treatment groups by EQ-5D, DIQI, and Psoriasis PASI, and the difference between treatment and placebo groups was significant by all measures. Shikiar et al. [33] confirmed that two treatment groups improved significantly greater than placebo measured using EQ-5D (*p* < 0.01), EQ-VAS (*p* < 0.01), and most SF-36 domains (*p* < 0.05), as well as DLQI. Another study [31] showed that EQ-5D and DLQI, PASI, PGA, EQ-VAS, and most SF-36 domains detected significant differences between responders and non-responders and DLQI was the most responsive with an effect size of 0.4 and EQ-5D had an effect size of 0.12, which was comparable to EQ-VAS and SF-36 domains. Weissi et al. [34] reported that after 2 weeks of therapy, scores improved significantly as shown by EQ-5D (by 11.5 %, *p* < 0.05), EQ-VAS (by 8.2 %, *p* < 0.001), PASI (by 26.2, *p* < 0.05), total body surface (by 20.4 %, *p* < 0.001) and another version of the PASI (i.e., SAPASI) (by 26.2 %, *p* < 0.05). Finally, Van de Kerkhof [23] showed that significant improvement was detected by EQ-VAS, Psoriasis Disability Index, and the pain/discomfort and anxiety/depression dimensions of EQ-5D although no statistical tests were reported.

### Acne

#### Known group analysis

In a case–control study, Klassen et al. [22] found that patients with acne reported higher proportions of problems for most EQ-5D dimensions than the general population, especially pain and anxiety.

#### Convergent validity

No study reported convergent validity in patients with acne.

#### Responsiveness

Klassen et al. [22] reported that after treatment the proportion of participants reporting a moderate problem on EQ-5D dimensions dropped greatly after treatment. EQ-5D utility values showed a significant change after treatment, which was consistent with SF-36 physical component summary score, and DLQI. A moderate effect size (0.44–0.53) for EQ-5D was reported whereas it was 0.98 for the DLQI, 0.3–0.5 for the SF-36 summary score, and 1.57 for the acne grades.

### Hidradenitis suppurativa

#### Known group analysis

For patients with hidradenitis suppurativa, Matusiak et al. [35] found that significant differences (*p* < 0.01) according to severity groups defined by Hurley’s classification groups were suggested by EQ-5D, EQ-VAS, DLQI, and the Beck Depression Inventory-Short Form.

#### Convergent validity

Moderate correlation (0.28 to 0.39, *p* < 0.05) was reported between EQ-5D with DLQI and EQ-5D with Functional Assessment of Cancer Therapy-Fatigue module (FACT-F) [35].

#### Responsiveness

No study reported responsiveness in patients with hidradenitis suppurativa.

### Hand eczema

#### Known group analysis

Among patients with hand eczema, Moberg et al. [36] suggested that EQ-5D and EQ-VAS significantly (*p* < 0.05) differ between groups defined according to whether they have hand eczema, as well as age and gender subgroups. The proportion of reporting any problems on the EQ-5D dimensions were also found for more groups with more severe disease but no statistical tests were reported.

#### Convergent validity

Moberg et al. [36] reported a strong correlation between EQ-5D and EQ-VAS among hand eczema patients.

#### Responsiveness

No study reported responsiveness in patients with hand eczema.

### Venous leg ulcers

#### Known group analysis

In patients with venous leg ulcers, Walters et al. [37] reported small effect sizes (less than 0.2) for the EQ-5D, EQ-VAS, SF-36, and Frenchay Activities Index (FAI) for patients grouped on the basis of their initial leg ulcer size, current ulcer duration, maximum ulcer duration, and age. On the other hand, the differences were statistically significant (*p* < 0.05) for the EQ-5D, EQ-VAS, FAI, and five subscales of SF-36 when groups were defined by whether they had none, moderate, or severe problems with mobility.

#### Convergent validity

Walters et al. [37] reported that EQ-5D achieved moderate-to-high correlation coefficients with SF-36 domains, the FAI, and the McGill Short Form Pain Questionnaire (SF-MPQ).

#### Responsiveness

Walters et al. [37] reported mixed results in a study of compression healing of venous leg ulcers in different settings. When grouped according to how well patients’ leg ulcers had healed at 3 months, a deterioration of health status over time was shown by the EQ-5D. Results from the SF-36 confirmed this, but conflicted with results from the VAS and the Short-form McGill Pain Questionnaire.

## Discussion and conclusions

This study aimed to systematically review and assess the validity, reliability and responsiveness of three GPBMs, namely EQ-5D, HUI3, and SF-6D in patients with skin diseases. There were no papers on the HUI3 and SF-6D, which met our inclusion criteria. The 16 studies included in the review provide useful information to assess the performance of EQ-5D skin disorders (see Table 3 for details).

**Table 3:** Overall performances of EQ-5D in skin conditions

	Conditions	Known group (severity)		Known group (case–control)		Known group (other)		Correlation	Responsiveness	
		Cons	Sig	Cons	Sig	Cons	Sig		Cons	Sig
Bansback et al. [24]	Psoriatic arthritis							✓		
Brodszky et al. [30]	Psoriatic arthritis					✓	✓	Strong		
Christophers et al. [25]	Plaque psoriasis and Psoriatic arthritis	✓	✓							
Daudén et al. [26]	Plaque psoriasis	✓	✓						✓	✓
Van de Kerkhof [23]	Plaque psoriasis			✓	N/R				✓	N/R
Luger et al. [27]	Plaque psoriasis	✓	✓						✓	✓
Reich et al. [28]	Plaque psoriasis			✓	✓				✓	✓
Revicki et al. [32]	Plaque psoriasis								✓	✓
Shikiar et al. [31]	Psoriasis							Moderate to strong	✓	✓
Shikiar et al. [33]	Psoriasis								✓	✓
Weiss et al. [29]	Psoriasis									
Weiss et al. [34]	Psoriasis			✓	✓			Moderate (sig)	✓	✓
Klassen et al. [22]	Acne			✓	✓				✓	✓
Matusiak et al. [35]	Hidradenitis suppurativa	✓	✓					Moderate		
Moberg et al. [36]	Hand eczema	✓	✓			✓	✓			
Walters et al. [37]	Venous leg ulcers	✓	N/R			✓	N/R		?	N/R

Empty cells indicate ‘no information is available’

*Cons* consistent evidence, *Sig* Statistically significant

✓ = Yes; ? = Mixed evidence; ✘ = No; N/R = no report

The findings were generally positive in terms of performance of EQ-5D. However, given the limited evidence for skin conditions apart from the plaque psoriasis and psoriatic arthritis for which most evidence was identified, this positive conclusion may not be generalizable to all skin conditions.

In the studies, EQ-5D was assessed in terms of ability to discriminate between groups or detect changes over time, and the convergence with other measures was taken as the evidence to support positive performance of EQ-5D. It is important to consider whether the measures of health that are being used for comparison are valid themselves. In addition, consideration must be given to the appropriateness of the clinical measure and the groups defined by it, and exogenous factors that may influence HRQL. For example, many studies included in our review used PASI, which is an accepted measure of psoriasis severity commonly used in studies of psoriasis and has been used as a measure of severity in development of clinical guidelines, but it does not measure HRQL. It is only an indicator for better or worse health.

It should be noted that the usefulness of the comparisons between HRQL measures can be limited by sample size, particularly as studies are usually not powered to detect differences according to preference-based measures. For instance, groups defined solely by the presence of a biomarker may have no impact on HRQL. Also, if patients have a number of co-morbidities, then these may have a greater impact on HRQL than the condition of interest.

We acknowledged that there was heterogeneity in the studies reviewed, in terms of study design, patient populations, and other HRQL measures used. However, in Table 3, each study was treated equally as a piece of evidence to assess the overall performance of health utility measure. This issue should be taken into account when interpreting the findings. Although there was a systematic search of literature across various databases, a limitation if the study is that the data extraction was undertaken mainly by one reviewer, with a sample of excluded papers checked by a second reviewer. Also, the review limited papers to those written in the English language, which might have excluded papers written in other languages. Further, the current review focused only on the three-level version of EQ-5D. Following increasing interest in and usage of the newly developed five-level EQ-5D measure, a similar review may be needed to examine how it performs in skin diseases. As demonstrated by a recent study [39], the five-level EQ-5D dimensions were good predictors of the psoriasis-specific DLQI and the SAPASI scores and it could differentiate severity groups defined by both DLQI and SAPASI scores. The study however also highlighted that including two additional dimensions specific to psoriasis increased the explanatory power of EQ-5D-5L to predict the DLQI and SAPASI scores, and this was also confirmed in the valuation study. The implications are that EQ-5D may perform satisfactorily for psoriasis, but bolt-on psoriasis-specific dimensions could potentially improve validity and responsiveness further.

It is surprising that no studies were found to provide sufficient evidence to assess performance of HUI3 and SF-6D in skin conditions. Also, no papers on reliability of any of the measures, including EQ-5D, were identified, which is a concern.

This is the first time information on the validity and responsiveness of GPBMs has been comprehensively reported and analyzed in skin disorders. Similar reviews using the same methodology have been undertaken for vision [14], mental health conditions [13, 16], hearing [38], and cancer [38]. We have established that EQ-5D is a responsive and valid measure of GBPM for use in patients with psoriasis and psoriatic arthritis. There was less evidence in patients with other skin conditions, but the limited evidence was generally supportive of EQ-5D. No evidence was found to assess the psychometric properties of HUI3 and SF-6D in patients with skin conditions, and no evidence on reliability was identified for any of the measures. This is a review of existing empirical studies on validity and responsiveness of GPBMs in patients with skin disorders. Empirical studies are needed to assess performance of HUI3 and SF-6D in patients with skin conditions, and to expand knowledge of EQ-5D in patients with other skin conditions apart from psoriasis and psoriatic arthritis.

## Appendix: Search strategy used in MEDLINE for the skin review

(Database: Ovid MEDLINE(R) In-Process & Other Non-Indexed Citations and Ovid MEDLINE(R) 〈1950 to Present〉).

1. [euroqol or euro qol or eq5d or eq 5d or eq-5d or (euro adj qol) or (eur adj qual) or (eq adj 5d)].mp. (2,151)
2. (hui3 or hui 3 or health utilities index mark 3 or health utilities mark three or hui III or huiIII).mp. (231)
3. (sf6D or sf 6D or short form 6D or shortform 6D or sf six D or sfsixD or shortform six D or short form sixD or sf-6d or 6d or 6-d or 6 dimension).mp. (4,538)
4. 1 or 2 or 3 (6,722)
5. Staphylococcal scalded skin syndrome.mp. or staphylococcal scalded skin syndrome/(414)
6. Impetigo.mp. or Impetigo/(1,457)
7. boil.mp. or Furunculosis/(1,278)
8. furunculosis.mp. (1,165)
9. Cutaneous abscess.mp. (66)
10. Cellulitis/or Cellulitis.mp. (8,369)
11. Acute lymphadenitis.mp. (30)
12. Pilonidal cyst.mp. (116)
13. Pyoderma/or Pyoderma.mp. (3,928)
14. Erythrasma.mp. or Erythrasma/(175)
15. Pemphigus/or Pemphigus.mp. (7,253)
16. Pemphigoid.mp. or Pemphigoid, Bullous/(4,942)
17. Dermatosis.mp. or Skin Diseases/(46,511)
18. Acantholysis/or Acantholytic disorder.mp. (660)
19. Dermatitis/or Dermatitis.mp. (59,308)
20. Eczema/or eczema.mp. (13,886)
21. prurigo.mp. or Prurigo/(1,207)
22. Pruritus.mp. or Pruritus/(13,152)
23. Lichen simplex chronicus.mp. or Neurodermatitis/(1,396)
24. Dyshidrosis.mp. (104)
25. Erythema intertrigo.mp. (2)
26. Pityriasis alba.mp. (79)
27. Papulosquamous.mp. (861)
28. Psoriasis.mp. or Psoriasis/(27,853)
29. Acrodermatitis/or Acrodermatitis continua.mp. (1,813)
30. Pustulosis.mp. (1,302)
31. Urticaria/or Urticaria.mp. (12,733)
32. erythema.mp. or Erythema/(2,5199)
33. Sunburn.mp. or Sunburn/(2,693)
34. Dermatitis, Phototoxic/(528)
35. Dermatitis, Photoallergic/or Photoallergic.mp. (700)
36. Solar urticaria.mp. (228)
37. Actinic keratosis.mp. or Keratosis, Actinic/(944)
38. Actinic reticuloid.mp. (139)
39. Cutis rhomboidalis nuchae.mp. (12)
40. Poikiloderma of Civatte.mp. (36)
41. Cutis laxa senilis.mp. (0)
42. Actinic granuloma.mp. (49)
43. Acne.mp. (11,465)
44. Rosacea.mp. or Rosacea/(2,084)
45. Vitiligo.mp. or Vitiligo/(4,053)
46. 5 or 6 or 7 or 8 or 9 or 10 or 11 or 12 or 13 or 14 or 15 or 16 or 17 or 18 or 19 or 20 or 21 or 22 or 23 or 24 or 25 or 26 or 27 or 28 or 29 or 30 or 31 or 32 or 33 or 34 or 35 or 36 or 37 or 38 or 39 or 40 or 41 or 42 or 43 or 44 or 45 (212,215)
47. 4 and 46 (60)
